# Determination of the performance of a novel diagnostic test for *Clostridioides difficile* toxins A and B using latent class analysis

**DOI:** 10.1128/jcm.01807-24

**Published:** 2025-03-31

**Authors:** Jeremy Li, Nandini Dendukuri, Yves Longtin, Alice Banz, Charles Frenette, Philippe Gervais, Mark A. Miller, Anne-Marie Bourgault, Noah L. Dawang, Vivian G. Loo

**Affiliations:** 1 McGill University 5620, Montreal, Quebec, Canada; 2 McGill University Health Centre Research Institute, Montreal, Quebec, Canada; 3 Jewish General Hospital, Montreal, Québec, Canada; 4 BioMérieux France, Marcy-Etoile, France; 5 McGill University Health Centre 54473, Montreal, Québec, Canada; 6 Institut universitaire de cardiologie et de pneumologie de Québec, Quebec, Quebec, Canada; 7 Université de Laval, Quebec, Quebec, Canada; Johns Hopkins University, Batimore, Maryland, USA

**Keywords:** *Clostridioides difficile*, multiple latent variable model, latent class analysis, single-molecule array

## Abstract

The diagnosis of *Clostridioides difficile* infection (CDI) remains challenging. Nucleic acid amplification tests (NAAT) targeting the *C. difficile* (CD) toxin B gene suffer from suboptimal specificity for CDI due to CD asymptomatic colonization. Enzyme immunoassays (EIAs) that detect the presence of CD toxins are more specific for CDI but suffer from low sensitivity. To address this challenge, assays detecting CD toxins were developed using single-molecule array (SIMOA) technology, which have much lower limits of toxin detection than conventional EIAs. In this study, stool specimens from 708 symptomatic patients were aliquoted for testing by cell cytotoxicity neutralization assay (CCNA), toxigenic culture, NAAT, conventional CD toxin EIA, and SIMOA CD toxin EIAs. Using latent class analysis, we calculated the sensitivity and specificity of each of these diagnostic tests for detecting, separately, the presence of CD bacterium, CD toxin gene, and CD toxin. We estimated that the prevalence of CDI in our cohort was 14% (95% credible interval [CI]: 0.11–0.17). While the specificity of NAAT for detecting the presence of CD toxin was 95% (95% CI: 0.94–0.97), its positive predictive value was poor due to the low prevalence of CDI. The specificity of the conventional CD toxin EIA for CDI was excellent, but the sensitivity was only 48% (95% CI: 0.41–0.55). In comparison, the sensitivities of the SIMOA toxins A and B EIAs were 76% (95% CI: 0.67–0.84) and 77% (95% CI: 0.67–0.84), respectively, while maintaining excellent specificity. We conclude that SIMOA CD toxin EIAs are significantly more sensitive than conventional CD toxin EIAs.

**IMPORTANCE:**

*Clostridioides difficile* infection (CDI) is the most important infectious cause of hospital-associated diarrhea worldwide, but its diagnosis remains challenging. Nucleic acid amplification tests (NAATs) targeting the *C. difficile* (CD) toxin B gene have suboptimal specificity due to the presence of CD asymptomatic colonization, while enzyme immunoassays (EIAs) that detect the toxin itself are much more specific but are limited by low sensitivity. New assays for detecting CD toxins were developed using single-molecule array (SIMOA) technology, which have much lower limits of toxin detection than conventional EIAs, potentially improving the sensitivity of these conventional EIAs while remaining highly specific. In this study, we use latent class analysis to evaluate the sensitivity and specificity of different diagnostic tests for CD, including the novel SIMOA toxin assays, in detecting the different CD targets: the presence of CD bacterium, the presence of CD toxin gene, and the presence of CD toxin.

## INTRODUCTION

*Clostridioides difficile* (CD) is the most important cause of infectious healthcare-associated diarrhea in Canada and worldwide (1–3). The accurate and rapid diagnosis of *C. difficile* infection (CDI) is crucial for prompt, appropriate treatment, and prevention of transmission.

CD diagnostics remain challenging and rely on positive laboratory assays in patients presenting with compatible symptoms. Numerous assays are available, but assays that detect the presence of toxins have been reported to be the most specific for the diagnosis of CDI. While the cell cytotoxicity neutralization assay (CCNA) is most correlated with clinical outcomes and is considered to be the best test for CDI (4), it is time-consuming, difficult to standardize, and not routinely performed in clinical laboratories. Many laboratories use nucleic acid amplification tests (NAAT) to detect the toxin B gene in stool for the diagnosis of CDI (5). However, NAAT is not specific for the diagnosis of CDI because asymptomatic colonization of CD (CD-AC) with a toxigenic strain can occur (6). Conversely, enzyme immunoassays (EIAs) that detect CD toxin are highly specific for CDI, but suffer from poor sensitivity, reported to be in the 33-65% range (7–9).

To address this challenge, novel quantitative assays for CD toxins A and B were developed, using single-molecule array (SIMOA) technology. It has previously been shown that SIMOA technology is able to detect very low quantities of analyte. Preliminary data obtained with SIMOA CD toxins A and B enzyme-linked immunosorbent assays show a 1,000-fold increase in sensitivity with limits of detection of 0.45 and 1.5 pg/mL, respectively (10). In comparison, traditional qualitative CD toxin EIAs have limits of detection that range from 0.8 to 2.5 ng/mL (11).

In this study, stool specimens submitted for CD testing at our clinical laboratory were tested by multiple diagnostic methods: CCNA, toxigenic culture, NAAT, glutamate dehydrogenase (GDH) EIA, conventional qualitative toxins A and B EIA, as well as the novel SIMOA toxins A and B EIA. We estimate the sensitivity and specificity of each of these diagnostic tests for CDI (the target condition of interest) using a multiple latent variable model (12). This statistical model can be used to estimate the sensitivity and specificity of diagnostic tests without assuming the existence of a gold standard. The multiple latent variable model extends the traditional latent class model with two latent classes (target condition positive and target condition negative) by recognizing that the observed diagnostic tests are measuring multiple related underlying latent variables besides the target condition. This model is well suited for studying CD testing because diagnostic tests for CD do not all measure true CDI. Only CCNA and CD toxin EIAs measure the presence of toxin in stool and are thought to be the most specific for CDI (4). NAAT and toxigenic culture detect the presence of toxigenic strains of CD, including cases of CD-AC when there may be no detectable toxin present (6). GDH antigen EIA and CD culture are less specific still and detect colonization even by non-toxigenic strains of CD (6). We use the multiple latent variable model to separately estimate the sensitivity and specificity of each of these diagnostic tests in detecting the different CD targets: the presence of CD bacterium, the presence of CD toxin gene, and the presence of CD toxin.

## MATERIALS AND METHODS

From March 15, 2018, to August 31, 2020, patients 18 years or older who had stool samples ordered for CD testing were recruited to participate in the study from two university-affiliated hospitals in Montreal, Quebec, Canada: McGill University Health Centre and Jewish General Hospital. Participants must have had at least three liquid stools (Bristol score 5–7) in 24 h without an alternative explanation to be eligible. The microbiology laboratory routinely rejected formed stools. Patients were excluded from the study if they had colostomies, received ≥24 h of specific therapy against CDI, or if they had already participated in the study. We did not specifically exclude patients who were on laxatives, but nurses were instructed not to submit stools for CD analysis if patients were receiving laxatives.

Stool specimens from participants were aliquoted for testing by CCNA, toxigenic culture, GDH EIA, NAAT, conventional CD toxins A and B EIA, and SIMOA toxins A and B EIA. Stool samples were processed within 24 h of collection or multiple aliquots of stool samples were frozen only once at −80°C prior to testing to avoid multiple freeze-and-thaw cycles.

CCNA and toxigenic culture were performed as per standard methods (13). For CCNA, stool was centrifuged, filtered, and then inoculated onto MRC-5 cells on microwells using two dilutions of the filtrate. Positive results with characteristic cytopathic changes were confirmed by neutralization with CD antitoxin (Techlab Inc., Blacksburg, VA). For toxigenic culture, stool specimens were subjected to an alcohol shock procedure and then inoculated onto cycloserine-cefoxitin fructose agar supplemented with 5% horse blood and 0.1% taurocholate and incubated for 48 h. Isolates were confirmed to be CD by matrix-associated desorption/ionization time-of-flight using the VITEK MS (bioMérieux, Montreal, Canada). CD isolates were then assayed for toxin B production by CCNA.

Testing was performed as per the manufacturer’s instructions for NAAT using the Roche cobas Cdiff Test (Roche Diagnostics, Basel, Switzerland). Testing for GDH and toxins A and B were performed using Vidas GDH and Vidas qualitative toxins A and B EIA (bioMérieux, Montreal, Canada) as per the manufacturer’s instructions. The SIMOA assays were performed by bioMérieux. The thresholds at which the SIMOA toxins A and B quantitative EIA were interpreted as positive were 22.1 and 18.8 pg/mL, respectively, based on previous research by Banz et al. (14).

Patients were considered to have CDI if they met one of the following criteria: diarrhea with at least three liquid to semi-liquid stools in 24 h without any other apparent etiology, or toxic megacolon with a positive CD assay, a change in stool characteristics from baseline without apparent cause in a patient known for chronic diarrhea with a positive CD assay, endoscopic evidence of pseudomembranous colitis, or histopathologic evidence of CD colitis (15). Two certified infectious disease physicians or nurse practitioners independently adjudicated whether patients had CDI or colonization. The adjudicators had access to all clinical information including results of NAAT. However, they were blinded to CCNA, qualitative CD toxin EIA, CD toxigenic culture, and SIMOA toxin EIA results. Disagreements were resolved by another infectious disease physician. In addition, for each death, two physicians judged independently whether CDI was an attributable cause, a contributory cause, or unrelated to the cause of death. In the case of a disagreement, resolution was achieved by a third infectious disease physician.

This study was approved by the institutional review board at the participating institutions. Informed consent was obtained from participants.

### Statistical analysis

**Fig 1:**
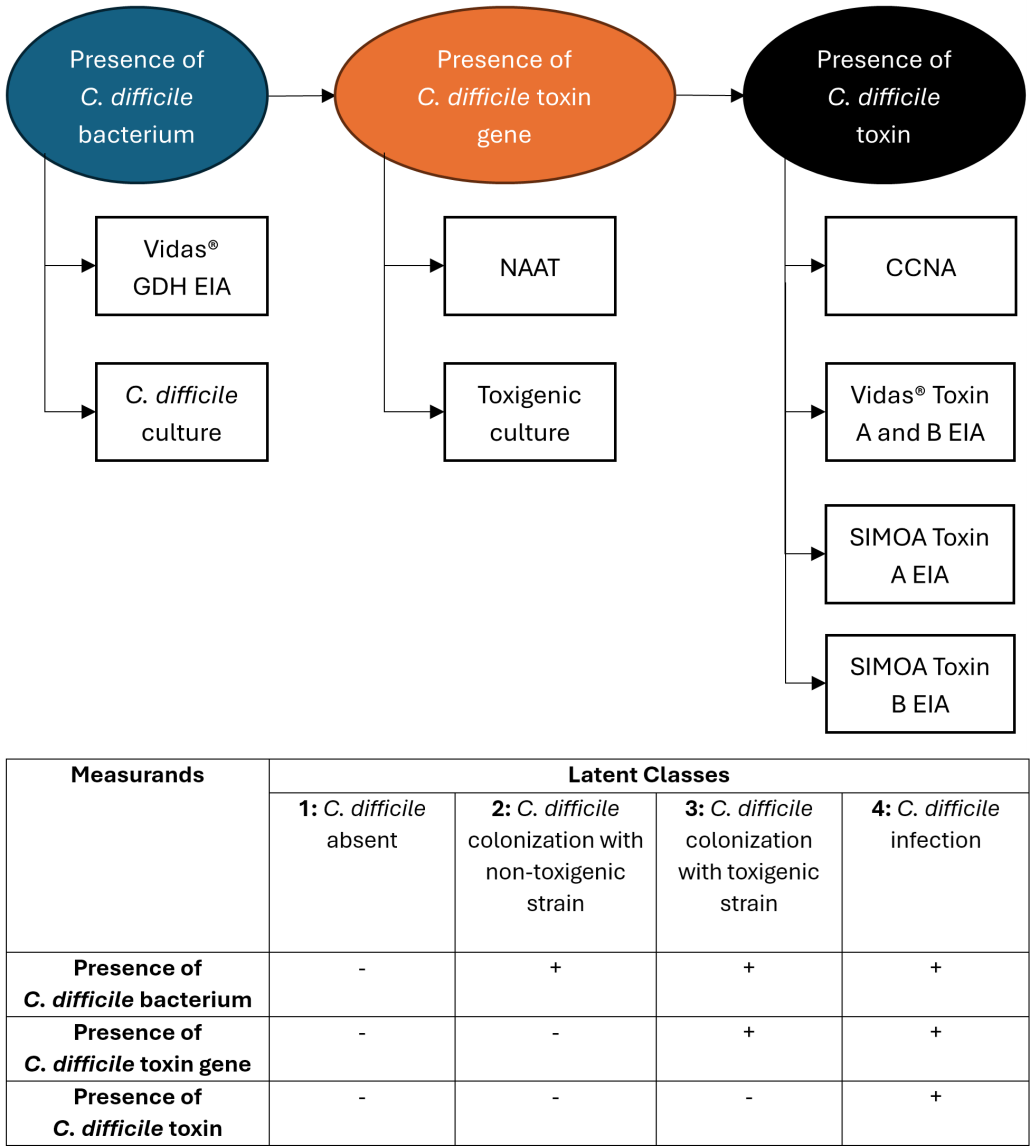
Association of each measurand with each latent class. The presence of *C. difficile* bacterium is indicated by a positive GDH EIA or *C. difficile* culture. The presence of the *C. difficile* toxin gene is indicated by NAAT or detection of an isolate of *C. difficile* by culture and confirmation of toxigenicity by subsequent CCNA (toxigenic culture). CCNA, qualitative toxin EIA, and SIMOA toxins A and B EIA detect the presence of *C. difficile* toxin. CCNA, cell cytotoxicity neutralization assay; EIA, enzyme immunoassay; GDH, glutamate dehydrogenase; NAAT, Nucleic acid amplification test; SIMOA, single-molecule array.

A multivariable latent class model was constructed. Figure 1 illustrates the association between each observed test (represented by rectangles) and its measurand (represented by ovals), and the association between the measurands and the target condition of CDI. Combinations of the measurands resulted in four latent classes: CD absent, CD colonization (bacteria present), CD colonization with toxigenic strain (toxin gene present), and CDI (toxin present). The association of each measurand with each latent class is shown in the table in Fig. 1. Clinician assessment of CDI is not used in the latent class model for the purpose of estimating the sensitivity and specificity of these diagnostic tests.

We introduced a single random effect to account for the dependence between multiple test results observed on the same subject. In the context of our latent class model, to avoid biased estimates of test accuracy, it is important to adjust for the dependence that arises conditional on the burden of the patient’s infection (16).

We used a Bayesian approach with non-informative prior distributions to estimate the prevalence of the four latent classes and the sensitivity and specificity of each test with respect to the target condition and other measurands. The model was fit using all available data, including those from subjects with some missing test results. The missing results were imputed. More technical details on the model, the program, and model checking appear in the supplementary material. Bayesian inference was carried out in the R software environment using the rjags package (17, 18).

Finally, we calculated the sensitivity, specificity, positive predictive value, and negative predictive value of the SIMOA toxins A and B EIAs, as well as NAAT, by comparing them directly to CCNA as a reference method.

## RESULTS

A total of 1,000 patients were approached to participate in the study at the two study sites. Of the patients who were approached, 292 patients declined to participate, and the remaining 708 patients were enrolled in the study. Stool specimens from these 708 patients were collected and aliquoted for testing. Baseline characteristics of the 708 enrolled patients are shown in Table 1.

**TABLE 1:** Characteristics of the 708 study patients among each latent class^*a*^^,^*^b^*^,^*^c^*^,^*^d^*^,^*^e^*

Variable	Overall	LC = 1	LC = 2	LC = 3	LC = 4
Number of patients	708	561	23	27	97
Age, mean (standard deviation)	63 (16.3)	63.3 (16.4)	65.6 (11.9)	54.6 (17.6)	63.1 (15.9)
Female, number (%)	351 (50%)	269 (48%)	10 (46%)	18 (66%)	54 (56%)
Charlson Comorbidity Index, mean (standard deviation)	3.8 (2.4)	3.8 (2.3)	3.5 (2.9)	3.2 (1.8)	3.7 (2.6)
History of CDI in the last 6 months, number (%)	28 (4%)	10 (2%)	2 (9%)	2 (8%)	14 (14%)
Laxative use on the day of specimen collection, number (%)	51 (7%)	36 (6%)	2 (7%)	3 (11%)	10 (11%)
Antibiotic use on the day of specimen collection, number (%)	407 (58%)	328 (59%)	11 (47%)	14 (53%)	54 (56%)
Antibiotic use 14 days prior to specimen collection, number (%)	497 (70%)	400 (71%)	15 (65%)	16 (60%)	66 (69%)
Highest WBC count (10^9^/L) within 2 days of specimen collection, mean (standard deviation)	11.2 (10.6)	11.4 (11.4)	9.9 (6.6)	9 (4.6)	11.2 (6.9)
Highest creatinine (µmol/L) within 2 days of specimen collection, mean (standard deviation)	121.2 (101.9)	119 (96.5)	111.9 (99.0)	132 (140.4)	133.4 (117.5)
Deaths (all causes)	51 (7.2%)	47 (8%)	1 (4%)	0 (0%)	3 (3%)
Deaths (due to CDI)	1 (0.1%)	0 (0%)	0 (0%)	0 (0%)	1 (1%)

^*a*^ WBC = white blood cell count, LC = latent class. Results within latent classes are rounded to the nearest whole number*.*

^*b*^ LC = 1, *C. difficile* absent.

^*c*^ LC = 2, *C. difficile* colonization with non-toxigenic strain.

^*d*^ LC = 3, *C. difficile* colonization with toxigenic strain.

^*e*^ LC = 4, *C. difficile* infection.

The positivity rate of the toxin B gene NAAT in our study was 16.2%. In comparison, the CCNA and toxigenic culture positivity rates were 9.7% and 15.5%, respectively. With regards to the EIAs, the positivity rates of the VIDAS GDH EIA and VIDAS toxins A and B EIA were 21.6% and 7.1%, respectively. The positivity rates of the SIMOA toxin A and B EIA were 12.5% and 11.8%, respectively. There were 36 (5%) stool specimens where either the SIMOA toxin A or B EIA was positive, but the CCNA was negative.

Table 2 shows the most common patterns of test results observed in our study data. Not all unique patterns of results are represented in this table. Of the 708 stool specimens, 488 specimens (69%) tested negative for CD by every diagnostic test. Thirty-one specimens (4.4%) tested positive for CD by every diagnostic test. Twenty-three specimens (3.2%) tested positive by GDH EIA, NAAT, and toxigenic culture, but tested negative for toxin by both VIDAS and SIMOA toxin EIAs as well as by CCNA. In another 17 specimens (2.4%), CD was isolated from culture, and GDH EIA was positive, but all other diagnostic tests, including NAAT and toxigenic culture, were negative. Other combinations of test results accounted for the remaining 154 specimens (22%), usually representing discordant results in one or more tests. For each pattern of results, we estimated the probability of it belonging to each of the four latent classes. For each pattern of results, we also listed the number of patients that met the clinical criteria for CDI. Estimates of the prevalence of each of the four latent classes are shown in Table 3.

**TABLE 2:** The most common patterns of test results observed in the study^*a*^

Detects CD bacterium		Detects CD toxin gene		Detects CD toxin				Frequency	Positive clinical diagnosis, *n* (%)	Probability of each latent class (median values)			
GDH EIA	CD culture	Toxigenic culture	NAAT	Toxins A and B EIA	SIMOA toxin A EIA	SIMOA toxin B EIA	CCNA			*C. difficile* absent	*C. difficile* colonization with non-toxigenic strain	*C. difficile* colonization with toxigenic strain	*C. difficile* infection
−	−	−	−	−	−	−	−	488	0 (0)	1	0	0	0
+	+	+	+	+	+	+	+	31	31 (100)	0	0	0	1
+	+	+	+	−	−	−	−	23	23 (100)	0	0	0.87	0.13
+	+	−	−	−	−	−	−	17	0 (0)	0	1	0	0
+	−	−	−	−	−	−	−	10	0 (0)	0.7	0.2	0	0.1
+	+	+	+	−	+	+	+	7	7 (100)	0	0	0	1
−	−	−	−	+	−	−	−	7	0 (0)	1	0	0	0

^*a*^ "+”, test interpreted as positive, “-”, test interpreted as negative.

**TABLE 3:** The estimated prevalence of each of the four latent classes in our model with 95% credible interval (CI)

Latent class	Prevalence (median, 95% CI)
*C. difficile* absent	0.79 (0.76–0.82)
*C. difficile* colonization with non-toxigenic strain	0.03 (0.02–0.05)
*C. difficile* colonization with toxigenic strain	0.04 (0.02–0.06)
*C. difficile* infection	0.14 (0.11–0.17)

The sensitivities and specificities of individual diagnostic tests with respect to each measurand (see Fig. 1), along with the 95% credible intervals (CIs) were calculated and are shown in Tables 4 and 5. Table 6 shows the sensitivity, specificity, positive predictive value, and negative predictive value of the SIMOA toxins A and B EIA and NAAT when compared directly to CCNA as a reference method.

**TABLE 4:** Estimates of the sensitivity of each test adjusting for conditional dependence (median, 95% credible interval) and with correction for sampling

	Sensitivity for the presence of ***C. difficile*** bacterium	Sensitivity for the presence of ***C. difficile*** toxin gene	Sensitivity for the presence of ***C. difficile*** toxin
Culture	0.91 (0.87–0.94)	0.92 (0.87–0.94)	0.92 (0.87–0.96)
VIDAS GDH	1.00 (0.98–1.00)	1.00 (0.98–1.00)	1.00 (0.98–1.00)
NAAT	0.76 (0.73–0.78)	0.90 (0.85–0.94)	0.90 (0.84–0.94)
Toxigenic culture	0.75 (0.72–0.78)	0.88 (0.83–0.90)	0.88 (0.83–0.91)
SIMOA A	0.50 (0.48–0.52)	0.59 (0.56–0.62)	0.76 (0.66–0.83)
SIMOA B	0.51 (0.49–0.53)	0.61 (0.57–0.63)	0.77 (0.67–0.84)
CCNA	0.47 (0.44–0.48)	0.55 (0.52–0.58)	0.71 (0.61–0.77)
VIDAS toxin EIA	0.33 (0.30–0.35)	0.38 (0.34–0.41)	0.48 (0.41–0.55)

**TABLE 5:** Estimates of the specificity of each test adjusting for conditional dependence (median, 95% credible interval) and with correction for sampling

	Specificity for the presence of *C. difficile* bacterium	Specificity for the presence of *C. difficile* toxin gene	Specificity for the presence of *C. difficile* toxin
Culture	1.00 (1.00–1.00)	0.96 (0.96–0.97)	0.93 (0.92–0.94)
VIDAS GDH	0.99 (0.98–1.00)	0.95 (0.94–0.96)	0.91 (0.90–0.93)
NAAT	0.99 (0.99–0.99)	0.99 (0.99–0.99)	0.95 (0.94–0.97)
Toxigenic culture	1.00 (1.00–1.00)	1.00 (0.99–1.00)	0.96 (0.95–0.98)
SIMOA A	0.98 (0.97–0.98)	0.98 (0.97–0.98)	0.98 (0.97–0.98)
SIMOA B	0.99 (0.99–0.99)	0.99 (0.98–0.99)	0.99 (0.98–0.99)
CCNA	1.00 (1.00–1.00)	1.00 (1.00–1.00)	1.00 (1.00–1.00)
VIDAS toxin EIA	0.99 (0.98–0.99)	0.98 (0.98, 0.98)	0.99 (0.98–0.99)

**TABLE 6:** The sensitivity, specificity, positive predictive value (PPV), and negative predictive value (NPV) of the SIMOA toxins A and B EIA and NAAT when compared directly to CCNA as a reference method

	SIMOA toxin A EIA	SIMOA toxin B EIA	NAAT
Sensitivity	0.84 (95% CI: 0.73–0.92)	0.82(95% CI: 0.71–0.90)	0.93 (95% CI: 0.84–0.98)
Specificity	0.95 (95% CI: 0.93–0.97)	0.96 (95% CI: 0.94–0.97)	0.92 (95% CI: 0.90–0.94)
PPV	0.67 (95% CI: 0.58–0.75)	0.70 (95% CI: 0.61–0.78)	0.56 (95% CI: 0.49–0.62)
NPV	0.98 (95% CI: 0.97–0.99)	0.98 (95% CI: 0.97–0.99)	0.99 (95% CI: 0.98–1.00)

## DISCUSSION

In our study, we confirmed the phenomenon of CD colonization, finding that the rate of CDI in our study was 14% (95% CI: 0.11–0.17), and the rate of CD colonization without toxin production was 7% (95% CI: 0.04–0.10). The latter group of patients may have had diarrhea for a reason other than CDI. Existing literature on CD-AC in hospitalized patients reports asymptomatic colonization rates of between 7% and 18% (19).

Our study population consisted only of patients for whom CDI was suspected. Our laboratory also rejects formed stool for CD testing. Therefore, we expect the proportion of CDI to CD colonization to be higher, and we expect fewer false positives from NAAT, toxigenic culture, and GDH EIA for the detection of CDI than if a random sample of hospital patients were selected. Even among patients for whom there is clinical suspicion of CDI, the rate of CDI in our study was only 14%.

We found that the sensitivity of NAAT and toxigenic culture for detecting the presence of toxigenic CD is approximately 90%. Concerns have previously been raised about the imperfect specificity and low positive predictive value of NAAT for the detection of CDI, due to the detection of colonization by toxigenic strains that are not actively producing toxin. In our study, the specificity of NAAT for detecting the presence of toxigenic strains of CD is 99% (including asymptomatic colonization), and the specificity of detecting true CDI (with toxin production) was only slightly lower at 95%. These results are close to estimates previously reported in the literature (20, 21).

We also demonstrated that, while the specificity of CCNA for detecting CDI was near 100%, the sensitivity was only 71%. Specifically, of the 97 stool specimens in which toxin was detected by either SIMOA toxin A or B EIA, only 61 (63%) were positive by CCNA. The sensitivity of SIMOA was also not perfect. Of the 69 stool specimens positive for toxin by CCNA, only 56 (81%) were positive by SIMOA toxin B assay.

In our model, the presence of CD toxin necessarily implied the presence of CD bacteria. Since the GDH EIA was 100% sensitive for detecting the presence of bacteria, it was therefore also 100% sensitive for detecting CD toxin.

Our research also confirmed the poor sensitivity of the conventional CD toxins A and B EIA, finding that its sensitivity was 48% (95% CI: 0.41–0.55), in agreement with what was previously reported in the literature (7–9). The sensitivity of the novel SIMOA toxins A and B EIAs was 76% and 77%, respectively, which is substantially higher than conventional qualitative EIAs, and better than our estimates for the sensitivity of CCNA. As expected, the specificities of all of the toxin immunoassays for CDI were excellent, approaching 100%.

Existing research has shown that CCNA is superior to toxigenic culture in predicting clinically relevant CD disease, that NAAT has poor positive predictive value for diagnosing CDI (4), and that the detection of CD toxin by CCNA is more predictive of CDI than detection of CD toxin gene by NAAT (22). Our model, in comparing the results of CCNA against other highly sensitive tests that detect very small amounts of CD toxin, found that the sensitivity of CCNA was only 71% (95% CI: 0.61–0.77). One explanation for this finding is that many NAAT-positive patients produce small amounts of toxin, detected by the novel SIMOA EIA, which would not otherwise be detected by conventional CD toxin EIA or CCNA. This finding highlights that toxin detection is highly dependent on laboratory methodology. One limitation of our model is that the detection of any amount of CD toxin was assumed to indicate CDI. However, it is questionable if such small amounts of toxin are clinically significant and may reflect colonization. Another possibility is that cross-reactivity in the CD Toxin EIAs to non-CD antigens is creating false positive and discordant results between these assays, thereby decreasing the calculated sensitivity of all toxin-specific assays in our model.

In our study, NAAT had the best overall test characteristics, with sensitivity of 90% (95% CI: 0.84–0.94) and specificity of 95% (95% CI: 0.94–0.97) as determined by our latent class model. Results are similar when comparing directly to CCNA as a reference method (Table 6). However, due to the low prevalence of CDI even among patients for whom CDI is clinically suspected, the positive predictive value of a positive NAAT was poor, as previously reported (4). In our study, of the 115 stool specimens that tested positive for NAAT, only 64 (56%) were positive for CCNA. In circumstances such as these, a more specific test may be desired. However, in the situation of having positive tests by CD culture, CD toxigenic culture, GDH, and NAAT and negative toxin tests by all assays, the probability of having CDI was 13%. This finding is supported by the study of Polage et al., in which 13 (8%) out of 61 patients who had a positive NAAT test and negative toxin test were retested within 14 days of the first test and were found to have toxin detected, indicative of CDI (23).

Based on our multiple latent variable model, both the SIMOA toxins A and B EIAs had superior sensitivity compared to conventional CD toxin EIA, perhaps even exceeding that of the reference CCNA method, while maintaining specificity approaching 100%. This assay has a much shorter turnaround time than CCNA and is much easier to standardize for a clinical laboratory. The positivity rates of the SIMOA toxins A and B EIAs were 12.5% and 11.8%, respectively, compared to 16.2% for NAAT, 9.7% for CCNA, and 7.1% for conventional CD toxin EIA. However, our research also showed that the sensitivity of the SIMOA toxins A and B EIAs may still be too low to be used as a standalone test and should be combined with a more sensitive test, such as NAAT or GDH EIA. Moreover, it has been reported that toxin concentrations, as measured by SIMOA, did not differentiate an individual with CDI from one with colonization (24). Further research is necessary to determine if these novel assays are as predictive of CDI as CCNA.

Our study is unique in that it used a multiple latent variable analysis to examine the performance of different CD laboratory assays on a very large sample. Previous analyses use models with two latent classes (25). The advantage of the multiple latent variable model is that it allows us to distinguish between the accuracy of each test with respect to its measurand versus the target condition. This would not be possible with a two-class model. Moreover, labeling the two latent classes would be challenging given the different measurands involved and the different numbers of tests for each measurand. We expect that each test has better accuracy with respect to its measurand. For example, whereas CD culture has excellent specificity for detecting *C. difficile* bacteria, its specificity for detecting the CD toxin gene or CD toxin is lower. More generally, the advantage of latent class analysis is that no test is arbitrarily assumed to have perfect accuracy. Rather, the sensitivity and specificity of all tests are modeled thus allowing us to take advantage of the results of all available tests to determine an individual’s probability of CDI.

Furthermore, we showed that toxin detection by conventional EIA has limited sensitivity and should not be the only determinant of whether the patient has CDI. A highly performant test for CDI with high sensitivity and specificity is still lacking, making the diagnosis of this disease difficult even with the many assays available. The diagnosis of CDI cannot solely rely on laboratory assays to distinguish between CD colonization and CDI as the assays are imperfect. The diagnosis of CDI still requires validation of clinical symptoms in combination with laboratory confirmation.

## SUPPLEMENTAL MATERIAL

Supplemental material jcm.01807-24-s0001.docx Description of the statistical model used to perform the latent class analysis, links to code, model fit statistics and plots, and ribotyping data.

ASM does not own the copyrights to Supplemental Material that may be linked to, or accessed through, an article. The authors have granted ASM a non-exclusive, world-wide license to publish the Supplemental Material files. Please contact the corresponding author directly for reuse.
