# Supplementary material for: Determination of the performance of a novel diagnostic test for Clostridioides difficile toxins A and B using latent class analysis
Source: J Clin Microbiol. 2025 Mar 31;63(5):e01807-24. doi: 10.1128/jcm.01807-24 (PMC12077097; doi:10.1128/jcm.01807-24)
Supplement: Supplemental material — Description of the statistical model used to perform the latent class analysis, links to code, model fit statistics and plots, and ribotyping data. [file jcm.01807-24-s0001.docx]

Supplemental Material

Likelihood function and prior distributions used in the Multiple Latent Variable Model

- *Notation:*

Let T1-T8 denote the 8 diagnostic tests as follows:

Test 1: SIMOA A

Test 2: SIMOA B

Test 3: CCNA

Test 4: VidasGDH

Test 5: Vidas Toxin

Test 6: NAAT

Test 7: C.difficile culture

Test 8: Toxigenic culture

Let y_ij_ denote the result of the j^th^ test on the i^th^ subject, j=1,…,8 and i=1,…,708.

- *Likelihood function:*

We assume that each test result follows a Bernoulli distribution and that the probability of a positive result is a mixture of probabilities across the four latent classes.

y_ij_ ~ Bernoulli(￼${prob}_{ij}$)

where ${prob}_{ij}$ = $\sum_{=1}^{4} \mathrm{pI}_{\mathrm{ikj}}*I(\mathrm{LC}_{i}=k)$

where $\mathrm{LC}_{i}$ is the latent class to which the i^th^ individual belongs, $\mathrm{pI}_{\mathrm{ikj}}$ is the probability of a positive result on for the i^th^ individual, on the j^th^ test, in the kth latent class and I(x) is the identity function such that I(x)=1 if x is true. The likelihood was adjusted to acknowledge that toxigenic culture could not be performed when *C. difficile* culture was negative.

Constraints were placed on the probabilities in each latent class according to Figure 1. For example, for the four tests that detect *C. difficile* toxin (Simoa A, Simoa B, CCNA and Vidas Toxin), the probability of a positive test is the same in latent classes 1-3, in which subjects do not carry any *C. difficile* toxin.

$${pI}_{i1j}=\Phi\left( a_{1j} +b_{1j}*r_{i} \right)$$

$${pI}_{i2j}={pI}_{i1j}$$

$${pI}_{n3j}={pI}_{n1j}$$

$${pI}_{i4j}=\Phi\left( a_{4j} +b_{4j}*r_{i} \right)$$

The probability of a positive test is a function of the severity of the infection in the i^th^ subject, represented by the random effect, $r_{i}$. The random effect $r_{i}$ is assumed to follow a standard normal distribution.

Similarly, constraints are introduced so that the two tests detecting the presence of *C. difficile* toxin gene have the same probability of a positive result in latent classes 1 and 2, as well as in 3 and 4. The two tests detecting the presence of *C. difficile* bacteria have the same probability of a positive test in latent classes 2, 3 and 4. The same random effect, $r_{i}$, also influences the probability of a negative result on all tests, thus setting up a dependence between the tests within each latent class.

- *Prior distributions:*

We used a Bayesian approach for inference. Therefore, a prior distribution was provided for each unknown parameter (summarizing any available knowledge about the unknown parameter external to the study). We used non-informative prior distributions for all parameters. In other words, the results were not influenced by any external information.

For the prevalence of the latent classes we used a Dirichlet(1,1,1,1) prior distribution. The equations above show that the probability of a positive test depends on parameters $a_{kj}$ and $b_{kj}$. . We assumed that $a_{kj}$ parameters followed a standard Normal(0,1) distribution and the $b_{kj}$ parameters followed a normal distribution truncated at 0 so that these parameters were always positive implying the impact of the severity $r_{i}$ on the test result was always positive.

Program and data

A program to prepare the data using R, a program to estimate the multiple latent class model using R and is available at this [link](https://github.com/nandinidendukuri/Cdifficile_LCA). The data used for the study is available on request.

rjags model

The rjags model used to estimate the multiple latent class model is available at this [link](https://github.com/nandinidendukuri/Cdifficile_LCA) under the sub-directory **rjags model.**

Convergence plots

We ran three different chains with randomly selected initial values. All three chains converged to the same solution for all parameters. The trace plots and density plots can be viewed at this [link](https://github.com/nandinidendukuri/Cdifficile_LCA) under the directory **Convergence Plots**.

Model fit statistics

- To examine the fit of the model we compared the observed and the expected frequency of subjects for each test pattern among subjects who had results on all tests. Table S1 (see following page) summarizes each pattern, its observed frequency and the median and 95% credible interval of the expected frequency. We found that the median expected values are close to the observed values and that the 95% credible intervals always included the observed value.
- As a second check of the model fit we calculated the correlation residuals comparing the observed and expected correlation for each pair of tests. Table S2 provides the median and 95% credible intervals for the correlation residuals. In all cases the median was close to 0 and the 95% credible interval included 0 suggesting that the model was satisfactorily able to capture any conditional dependence between the tests.

Identifiability

Latent class models are prone to non-identifiability, i.e. the presence of multiple solutions for the unknown parameters. We examined two commonly used conditions to check for identifiability:

- *Necessary condition for identifiability: This condition requires that the number of degrees of freedom exceeds the number of unknown parameters.* There are a total of 7 dichotomous tests. However, toxigenic culture can be positive only for those subjects who are culture positive. Thus, the total number of available degrees of freedom is 2^6+2^5 – 1 = 95. The total number of unknown parameters in the likelihood function is 3 prevalence parameters + 8*2 *a* parameters + 8*2 *b* parameters = 35, which is much lower than the number of degrees of freedom available.
- *Sufficient condition for local identifiability:* This condition requires that the determinant of the Jacobian of the transformation from the multinomial probability (for the joint distribution of the eight tests) to the 35 parameters of the latent class model is non-zero. This condition was verified using a program provided in the supplementary material under the directory **Local Identifiability**.

As the model is identifiable, we were able to obtain a unique solution using non-informative prior distribution for all parameters.

Ribotyping

Of the 708 stool specimens we tested, 134 (18.9%) were positive by CD culture and 114 (85%) were available for ribotyping. The ribotype distribution is shown in Table S3. The most common ribotype was 106 accounting for almost 20% of the isolates. The hypervirulent ribotype 027 only accounted for 5% of the isolates.

| Pattern #  Table S1: Observed and expected frequency of each observed patterns of test results | SIMOA A | SIMOA B | CCNA | VIDAS GDH | VIDAS TOXIN | NAAT | CULTURE | TOX_CULT | Observed  Frequency | Expected Frequency | | |
| --- | --- | --- | --- | --- | --- | --- | --- | --- | --- | --- | --- | --- |
|  |  |  |  |  |  |  |  |  |  | Median | 2.5% quantile | 97.5% quantile |
| 1 | 0 | 0 | 0 | 0 | 0 | 0 | 0 | 0 | 488 | 478 | 459 | 493 |
| 2 | 0 | 0 | 0 | 0 | 0 | 0 | 1 | 0 | 1 | 1 | 0 | 6 |
| 3 | 0 | 0 | 0 | 0 | 0 | 1 | 0 | 0 | 4 | 4 | 0 | 11 |
| 4 | 0 | 0 | 0 | 0 | 1 | 0 | 0 | 0 | 7 | 8 | 2 | 17 |
| 5 | 0 | 0 | 0 | 1 | 0 | 0 | 0 | 0 | 10 | 9 | 3 | 19 |
| 6 | 0 | 0 | 0 | 1 | 0 | 0 | 1 | 0 | 17 | 17 | 12 | 23 |
| 7 | 0 | 0 | 0 | 1 | 0 | 0 | 1 | 1 | 4 | 4 | 1 | 10 |
| 8 | 0 | 0 | 0 | 1 | 0 | 1 | 0 | 0 | 4 | 1 | 0 | 4 |
| 9 | 0 | 0 | 0 | 1 | 0 | 1 | 1 | 1 | 23 | 19 | 13 | 25 |
| 10 | 0 | 0 | 0 | 1 | 1 | 0 | 1 | 0 | 1 | 0 | 0 | 2 |
| 11 | 0 | 0 | 1 | 0 | 0 | 0 | 0 | 0 | 1 | 1 | 0 | 6 |
| 12 | 0 | 0 | 1 | 1 | 0 | 0 | 1 | 1 | 2 | 0 | 0 | 2 |
| 13 | 0 | 0 | 1 | 1 | 0 | 1 | 0 | 0 | 1 | 0 | 0 | 1 |
| 14 | 0 | 0 | 1 | 1 | 0 | 1 | 1 | 1 | 3 | 1 | 0 | 5 |
| 15 | 0 | 1 | 0 | 0 | 0 | 0 | 0 | 0 | 2 | 6 | 1 | 14 |
| 16 | 0 | 1 | 0 | 1 | 0 | 0 | 1 | 1 | 2 | 0 | 0 | 3 |
| 17 | 0 | 1 | 0 | 1 | 0 | 1 | 1 | 1 | 3 | 2 | 0 | 7 |
| 18 | 0 | 1 | 1 | 1 | 0 | 1 | 1 | 0 | 1 | 0 | 0 | 2 |
| 19 | 0 | 1 | 1 | 1 | 0 | 1 | 1 | 1 | 3 | 2 | 0 | 6 |
| 20 | 1 | 0 | 0 | 0 | 0 | 0 | 0 | 0 | 6 | 12 | 4 | 22 |
| 21 | 1 | 0 | 0 | 0 | 1 | 0 | 0 | 0 | 1 | 0 | 0 | 1 |
| 22 | 1 | 0 | 0 | 1 | 0 | 0 | 1 | 1 | 1 | 0 | 0 | 2 |
| 23 | 1 | 0 | 0 | 1 | 0 | 1 | 1 | 1 | 1 | 2 | 0 | 5 |
| 24 | 1 | 0 | 0 | 1 | 1 | 1 | 0 | 0 | 1 | 0 | 0 | 0 |
| 25 | 1 | 0 | 1 | 1 | 0 | 1 | 1 | 1 | 3 | 2 | 0 | 6 |
| 26 | 1 | 0 | 1 | 1 | 1 | 1 | 1 | 1 | 2 | 1 | 0 | 5 |
| 27 | 1 | 1 | 0 | 0 | 0 | 0 | 0 | 0 | 4 | 0 | 0 | 1 |
| 28 | 1 | 1 | 0 | 1 | 0 | 1 | 1 | 0 | 1 | 0 | 0 | 2 |
| 29 | 1 | 1 | 0 | 1 | 0 | 1 | 1 | 1 | 6 | 3 | 0 | 8 |
| 30 | 1 | 1 | 0 | 1 | 1 | 1 | 1 | 1 | 2 | 3 | 0 | 9 |
| 31 | 1 | 1 | 1 | 1 | 0 | 0 | 1 | 1 | 1 | 0 | 0 | 3 |
| 32 | 1 | 1 | 1 | 1 | 0 | 1 | 0 | 0 | 2 | 0 | 0 | 1 |
| 33 | 1 | 1 | 1 | 1 | 0 | 1 | 1 | 1 | 7 | 9 | 3 | 16 |
| 34 | 1 | 1 | 1 | 1 | 1 | 0 | 1 | 1 | 1 | 0 | 0 | 3 |
| 35 | 1 | 1 | 1 | 1 | 1 | 1 | 1 | 0 | 1 | 1 | 0 | 4 |
| 36 | 1 | 1 | 1 | 1 | 1 | 1 | 1 | 1 | 31 | 27 | 20 | 34 |

Table S2: Correlation residuals (difference between observed and expected correlation) for each pair of tests observed patterns of test results

| **Pairwise Correlation Residual** | **Median** | **2.5% quantile** | **97.5% quantile** |
| --- | --- | --- | --- |
| r12 | 0.06 | -0.04 | 0.17 |
| r13 | -0.02 | -0.12 | 0.09 |
| r14 | -0.01 | -0.08 | 0.07 |
| r15 | 0.02 | -0.09 | 0.14 |
| r16 | -0.03 | -0.11 | 0.07 |
| r17 | -0.03 | -0.1 | 0.06 |
| r18 | -0.04 | -0.12 | 0.05 |
| r23 | -0.03 | -0.13 | 0.08 |
| r24 | -0.01 | -0.08 | 0.07 |
| r25 | -0.03 | -0.14 | 0.1 |
| r26 | -0.04 | -0.12 | 0.06 |
| r27 | -0.01 | -0.08 | 0.07 |
| r28 | -0.03 | -0.11 | 0.06 |
| r34 | 0 | -0.06 | 0.08 |
| r35 | -0.01 | -0.13 | 0.13 |
| r36 | -0.02 | -0.1 | 0.07 |
| r37 | -0.01 | -0.08 | 0.06 |
| r38 | -0.02 | -0.1 | 0.07 |
| r45 | -0.01 | -0.09 | 0.08 |
| r46 | 0.01 | -0.05 | 0.07 |
| r47 | -0.02 | -0.06 | 0.03 |
| r48 | -0.02 | -0.06 | 0.03 |
| r56 | -0.03 | -0.13 | 0.08 |
| r57 | -0.01 | -0.09 | 0.08 |
| r58 | -0.02 | -0.12 | 0.08 |
| r67 | -0.04 | -0.09 | 0.02 |
| r68 | -0.05 | -0.11 | 0.02 |
| r78 | 0.01 | -0.03 | 0.05 |

r_pq_ is the correlation residual (i.e. difference between observed and expected correlation) between tests p and q.

Table S3: Ribotype distribution among 114 available CD isolates.

| Ribotype | Number of isolates (%) |
| --- | --- |
| 106 | 22 (19.2%) |
| 20 | 12 (10.5%) |
| 56 | 8 (7%) |
| 10 | 6 (5.3%) |
| 14 | 6 (5.3%) |
| 27 | 6 (5.3%) |
| 78 | 5 (4.4%) |
| 57 | 4 (3.5%) |
| 2 | 3 (2.6%) |
| 5 | 3 (2.6%) |
| 12 | 3 (2.6%) |
| 24 | 3 (2.6%) |
| 39 | 3 (2.6%) |
| 20 other ribotypes | 30 (26.3%) |
